# Can a single-port robot be safely used for robotic total gastrectomy for advanced gastric cancer? First experience using the da Vinci SP platform

**DOI:** 10.1093/gastro/goac023

**Published:** 2022-06-07

**Authors:** Hao Cui, Jian-Xin Cui, Ke-Cheng Zhang, Wen-Quan Liang, Shu-Yan Li, Jun Huang, Lin Chen, Bo Wei

**Affiliations:** Department of General Surgery, The First Medical Center, Chinese PLA General Hospital, Beijing, P. R. China; School of Medicine, Nankai University, Tianjin, P. R. China; Department of General Surgery, The First Medical Center, Chinese PLA General Hospital, Beijing, P. R. China; Department of General Surgery, The First Medical Center, Chinese PLA General Hospital, Beijing, P. R. China; Department of General Surgery, The First Medical Center, Chinese PLA General Hospital, Beijing, P. R. China; Foreign Languages College, Shandong University of Traditional Chinese Medicine, Jinan, Shandong, P. R. China; Department of General Surgery, The First Medical Center, Chinese PLA General Hospital, Beijing, P. R. China; School of Medicine, Nankai University, Tianjin, P. R. China; Department of General Surgery, The First Medical Center, Chinese PLA General Hospital, Beijing, P. R. China; School of Medicine, Nankai University, Tianjin, P. R. China; Department of General Surgery, The First Medical Center, Chinese PLA General Hospital, Beijing, P. R. China; School of Medicine, Nankai University, Tianjin, P. R. China

**Keywords:** gastric cancer, single port, robot, gastrectomy, da Vinci SP platform

## Abstract

**Background:**

Many studies have shown the operative feasibility and safety of robotic gastrectomy. Surgeons are pursuing single-port (SP) surgery to leverage the advantages of minimally invasive gastrectomy. The purpose of this study was to describe technical considerations and short-term outcomes from the first reported SP robotic total gastrectomy (RTG) using the da Vinci SP platform.

**Methods:**

A 75-year-old patient with a body-mass index of 19.8 kg/m^2^ and clinical stage III cancer (cT3N+M0) underwent SP RTG on 22 January 2022 at the Department of General Surgery, the Chinese PLA General Hospital. All procedures were performed successfully using the da Vinci SP robotic platform.

**Results:**

The SP RTG was successfully performed with D2 lymphadenectomy including No. 10 lymph-nodes dissection and extracorporeal Roux-en-Y anastomosis. Except for subcutaneous emphysema, no severe adverse events occurred during the operation. According to a visual analogue scale (VAS), the subjective feeling of post-operative pain was given a VAS score of 3 of 10 on Post-Operative Day 1 (POD 1), 1 of 10 on POD 3, and 1 of 10 on POD 7. We removed the gastric tube on POD 2 and advised sipping water, a liquid diet, and a soft diet on PODs 2, 4, and 6, respectively. The patient was discharged without any complications on POD 8.

**Conclusion:**

RTG is technically feasible and safe using the da Vinci SP robotic platform. To our knowledge, this is the first study using the da Vinci SP platform in RTG for advanced gastric cancer in elderly patients. To verify its superior operative outcomes, further clinical trials are needed.

## Introduction

Single-port (SP) robots, represented by the da Vinci SP robotic platform (Intuitive Surgical, Sunnyvale, CA, USA), have improved the minimal invasiveness of surgery. The platform allows convenient placement of three robotic arms and a camera into the abdomen via a 25-mm-diameter specialized instrument named the Entry Guide Cannula Insert and the multi-joint EndoWrist SP robotic arms can be flexibly rotated in the abdominal cavity to perform complicated operations in a narrow space. Compared with that of the conventional robotic platform, the operation performed by the SP robotic platform had several advantages, such as reduced post-operative pain, more cosmetic incisions, and easier laparoscopic exploration using the “RELOCATE” mode [1, 2].

For SP surgery, the SP robotic platform is more stable to operate than the SP laparoscope that is currently in common use [3]. Additionally, the snake-shaped robotic arm can effectively avoid the “chopstick effect,” reducing the number of surgical instrument collisions and the frequency of requiring additional ports to complete the surgery [4].

At present, the use of the da Vinci SP platform in urinary surgery [5] and gynecologic surgery [6] has been shown to be safe and feasible in several clinical studies. In the field of gastrointestinal surgery, the da Vinci SP platform has been reported to be used for radical resection for colon cancer [7, 8], transanal total mesorectal excision [9], and partial gastrectomy for gastric stromal tumors [10]. However, to our knowledge, no relevant studies have reported the application of the da Vinci SP robotic platform in radical total gastrectomy for gastric cancer. We now present the first case of robotic total gastrectomy (RTG) for locally advanced gastric cancer using the da Vinci SP platform, demonstrating its surgical safety and technical feasibility, and summarizing the surgical experience and personal perspectives to provide a clinical reference for further promotion of the system for this procedure.

## Methods

### Case presentation

**Figure 1.**
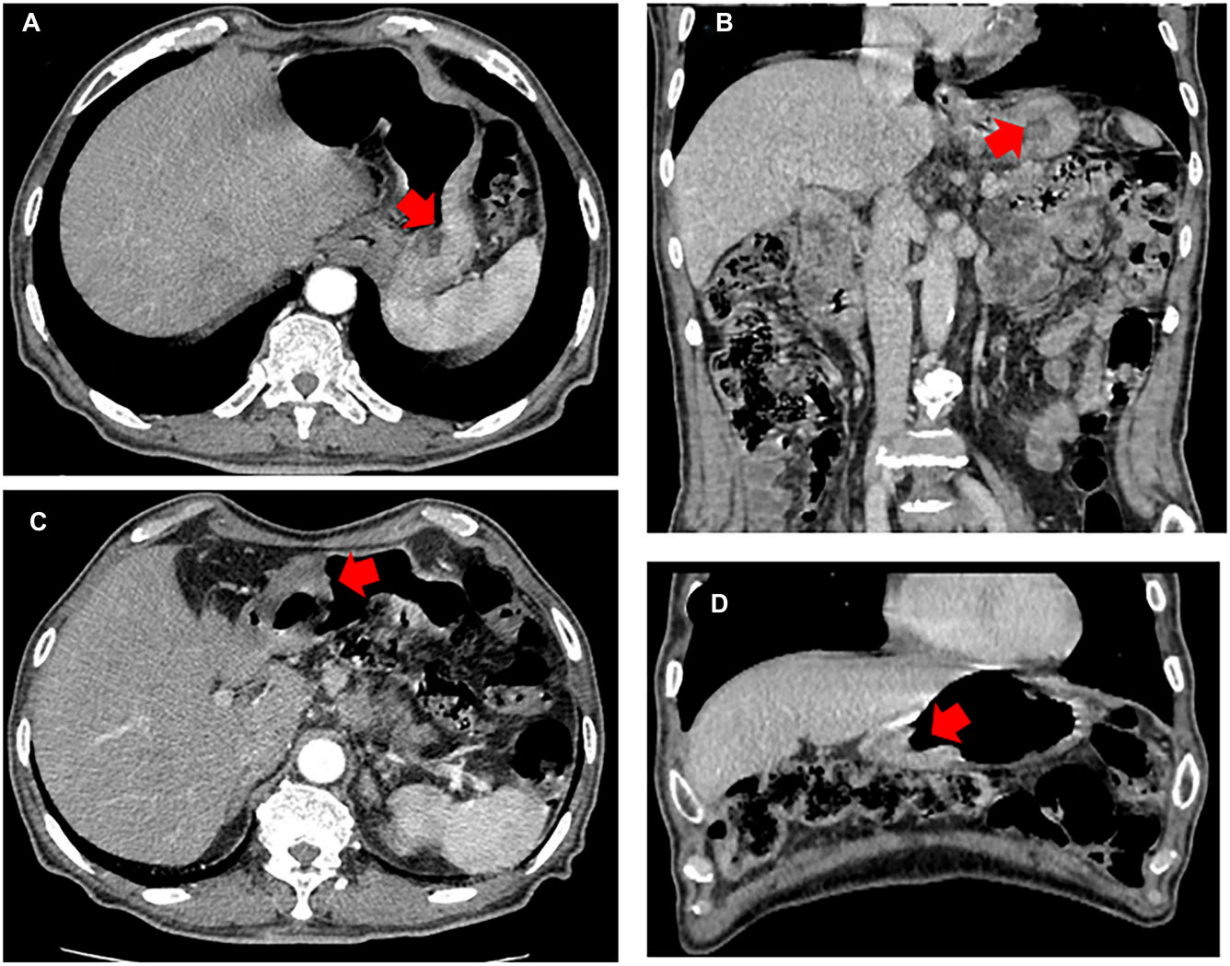
Preoperative abdominal computed tomography (CT) for gastric cancer. (A) Transverse section of the tumor located at the greater curvature of the upper stomach. (B) Coronal section of the tumor located at the greater curvature of the upper stomach. (C) Transverse section of the tumor located at the antrum of the stomach. (D) Coronal section of the tumor located at the antrum of the stomach. The red arrow represents the tumor location. (A colour version of this figure appears in the online version of this article.)

A 75-year-old male with a body-mass index (BMI) of 19.8 kg/m^2^ felt discontinuous epigastric discomfort for 4 months. At times he felt slight heartburn and belching, especially after eating. He had been diagnosed with type II diabetes 20 years prior and underwent inguinal hernia repair surgery in November 2021. Electronic gastroscopy revealed two separate ulcerative lesions: one at the greater curvature of the upper stomach measuring 4.0 × 2.5 × 1.0 cm and the other at the antrum of the stomach measuring 5.0 × 3.5 × 1.5 cm. Gastric adenocarcinoma was confirmed by pathological biopsy for each lesion. Enhanced abdominal computed tomography (CT) also showed gastric wall thickening and enlarged lymph nodes (LNs) in the lesser omental bursa, which is shown in Figure 1. The clinical stage was defined as stage III (cT3N+M0) according to the 8th edition of the American Joint Committee on Cancer staging manual.

Before surgery, a multidisciplinary consultation including the departments of general surgery, anesthesiology, cardiovasology, endocrinology, and pneumology was conducted to ensure perioperative safety. We systematically evaluated the patient’s physical status with an Eastern Cooperative Oncology Group score of 0, an American Society of Anesthesiologists scores of 2, and a Nutritional Risk Screening 2002 (NRS2002) score of 3, indicating that the patient could tolerate operative treatment. This study was approved by the ethics committee of the Chinese PLA General Hospital (Approval number: S2022-078–01) and we acquired informed consent from the patient before surgery.

### Procedures

#### Trocar placement and installation of the da Vinci SP platform

**Figure 2.**
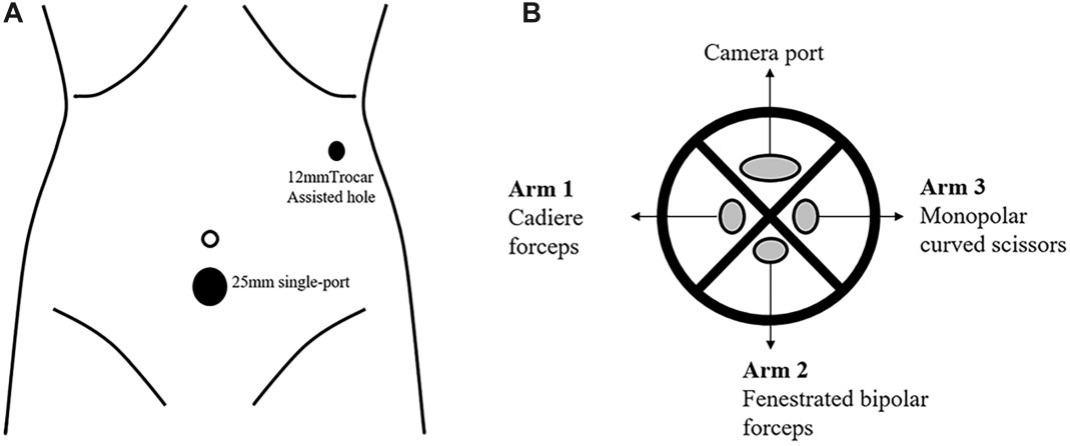
Port and robotic arm placement. (A) Schematic illustration of port placement of the single port and one assisted hole. (B) The platform of the single-port Entry Guide Cannula Insert including the camera port, cadiere forceps as Arm 1, fenestrated bipolar forceps as Arm 2, and monopolar curved scissors as Arm 3.

**Figure 3.**
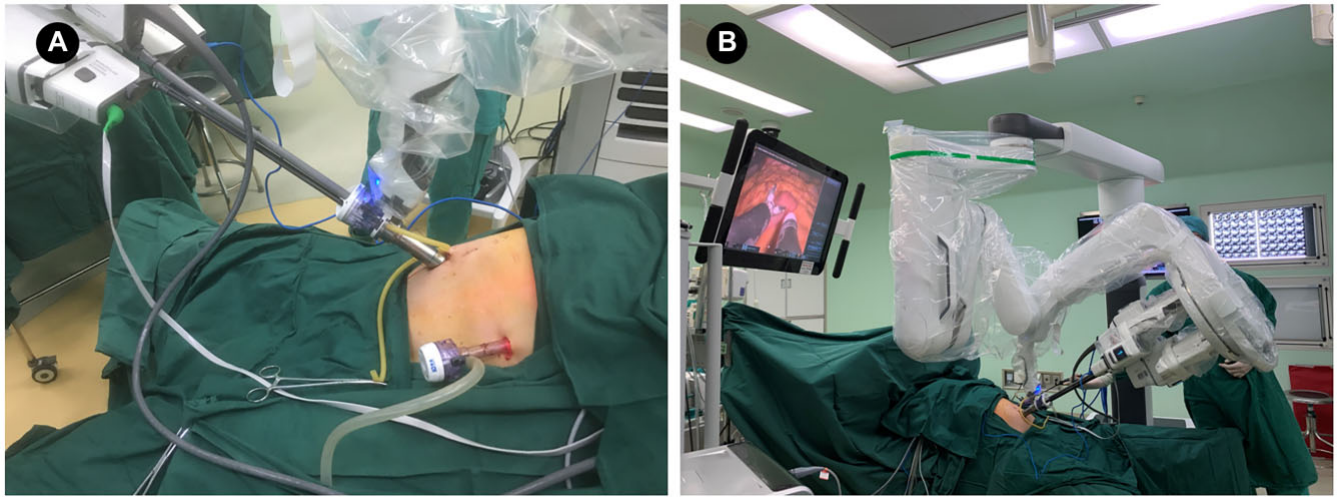
The placement of the da Vinci SP platform during single-port robotic total gastrectomy. (A) Composition of the single-port and trocar placement. (B) Surgical view of the console after installation of all surgical instruments.

The patient was placed in a reverse trendelenburg position under general anesthesia. The da Vinci SP patient cart base was placed on the left side of the patient. We chose to insert the 25-mm SP 2 cm below the umbilicus and the 12-mm assistive hole on the left anterior axillary line (Figure 2A). A 2.5-cm midline ventral incision was made below the umbilicus and the surgeons cut the tissue from the skin to the linea alba via electrocautery. After cutting the peritoneum, a 25-mm SP cannula was inserted and connected to the insufflation device. The entry guide for guiding the instrument and camera shafts through the cannula was then inserted into the cannula seal and the cannula fin was attached to the patient cart by pressing the cannula mount button. Then, three EndoWrist SP robotic arms and a camera were separately inserted into the entry guide; the camera was established through an upwards oval camera lumen, cadiere forceps were inserted to the right of the entry guide as Arm 1, fenestrated bipolar forceps were inserted opposite to the camera lumen as Arm 2, and monopolar curved scissors were inserted to the left of the entry guide as Arm 3 (Figure 2B). An external view of the trocar and da Vinci SP platform placement is shown in Figure 3.

#### Operative procedures

**Figure 4.**
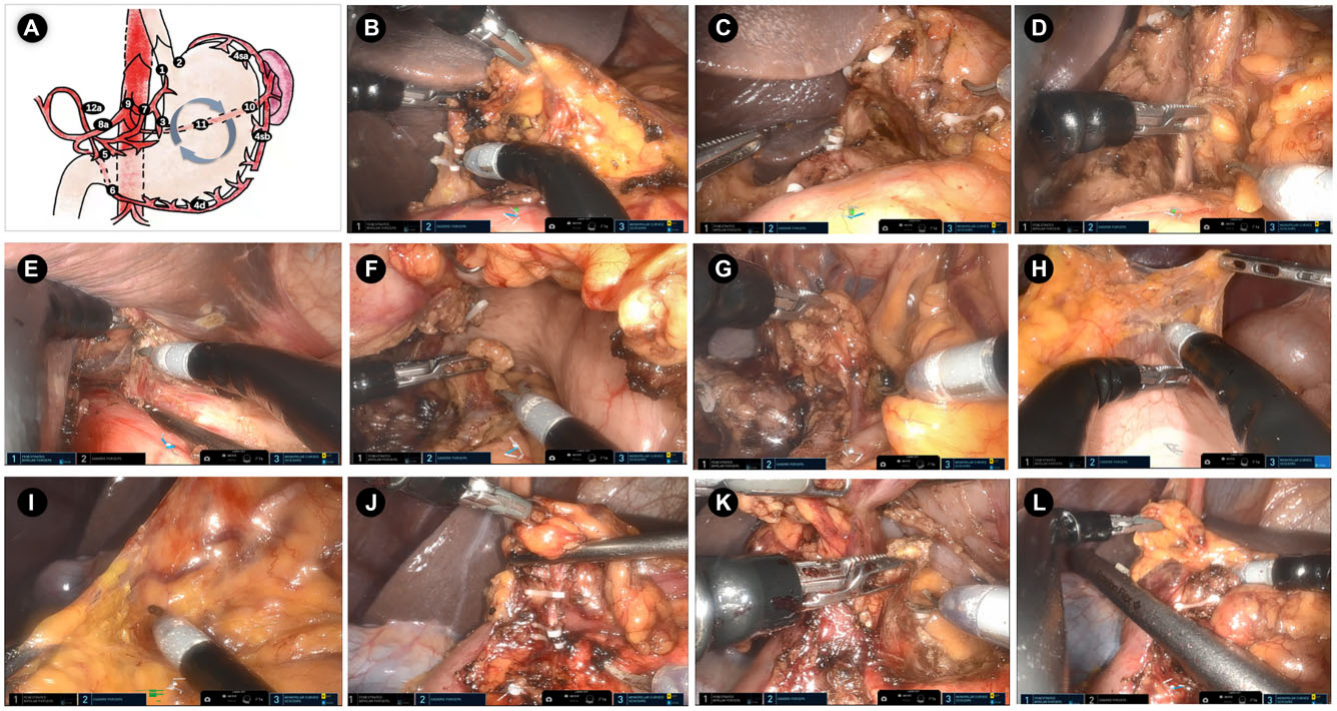
Scene of intracorporal operation during da Vinci SP robotic total gastrectomy. (A) Schematic diagram of clockwise D2 lymphadenectomy (gray cycle represents the order of lymphadenectomy). (B) Dissection of the No. 5, 12a, and 3 LNs. (C) Scene of supra-pancreatic regional LNs including the No. 7, 9, and 8a LNs. (D) Dissection of the No. 11p LNs. (E) Releasing the lower esophagus and dissecting the No. 1 and 2 LNs. (F) Dissection of the No. 11d and 10 LNs. (G) Dissection of the No. 4 LNs. (H) Cutting the gastrocolic ligament. (I) Exposing the fusion fascia that was located between mesogastrium and transverse mesocolon. (J) Dissection of the No. 6 LNs. (K) Exposure of the gastroduodenal artery (GDA). (L) Transecting the duodenum via an intracorporal linear stapler. LNs, lymph nodes.

We performed clockwise resection [11] to complete D2 lymphadenectomy including No. 10 LNs dissection, resulting in the successive dissection of LNs 5, 12a, 3, 7, 8a, 9, 11p, 1, 2, 4sa, 11d, 10, 4sb, 4d, and 6 in accordance with the Japanese Treatment Guidelines for Gastric Cancer 2018 (5th version) [12]. The spleen-preserving splenic hilar lymphadenectomy procedure was separated into three steps: (i) dissection of the LNs in the region of the trunk of the splenic artery; (ii) dissection of the LNs in the superior pole region of the spleen; and (iii) dissection of the LNs in the inferior pole region of the spleen. Then, 45-mm linear staplers were used to transect the esophagus and duodenum via the assistive hole after ensuring a sufficient cutting edge. Explicit images of the intracorporal LN dissection are shown in Figure 4 and a surgical video is shown in Supplementary Video 1. Arm 1 was used to expose the operating field, whereas Arm 2 and Arm 3 were mainly used to dissect the LNs and perform hemostasis. Because the patient developed subcutaneous emphysema and the tumor was located close to the dentate line, extracorporeal Roux-en-Y anastomosis via a 7-cm length epigastric incision was adopted after cutting the entire stomach in the abdominal cavity and we sutured the duodenal stump to avoid anastomotic-related complications. The specimen was removed from the epigastric incision and a flushable abdominal drainage tube was placed behind the esophagojejunal anastomotic stoma via the 12-mm assisted hole.

#### Perioperative outcomes

**Figure 5.**
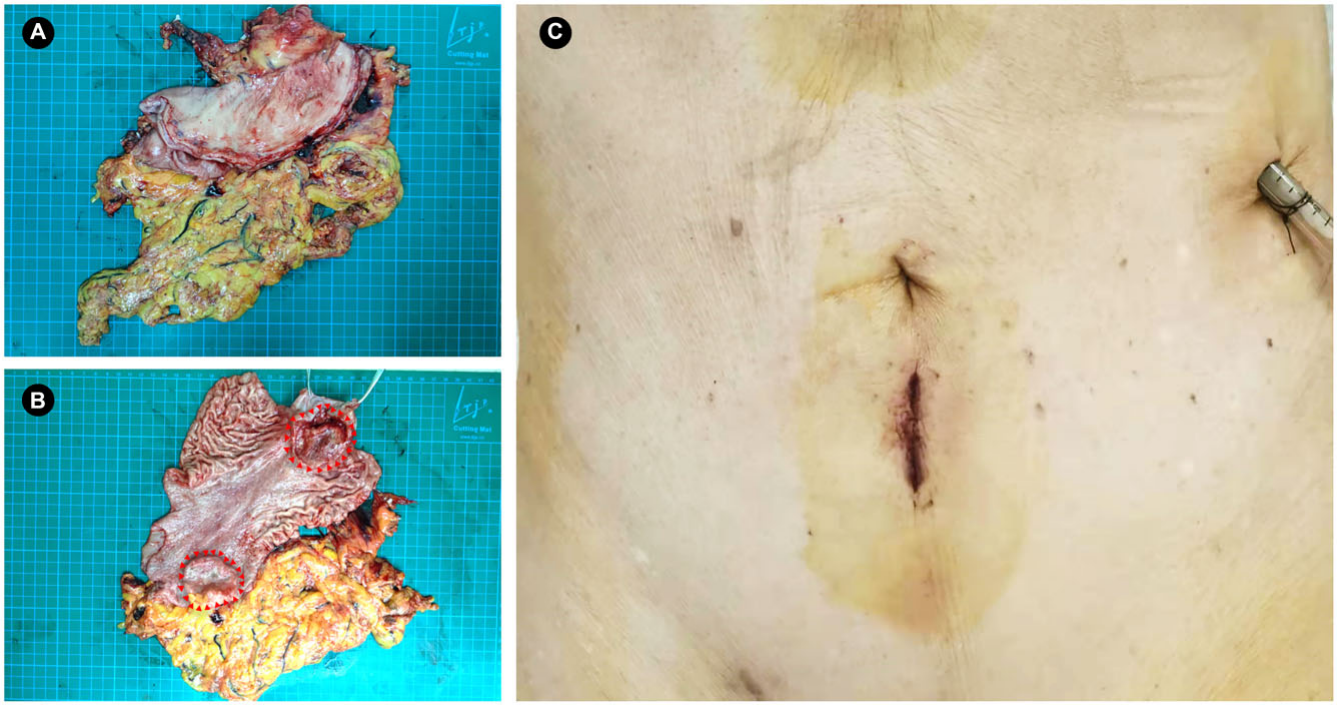
Specimen of the total stomach and final view of the surgical incision. (A) Overall view of the total stomach specimen. (B) Cutaway view of two gastric tumors that are surrounded by red arrows. (C) Final view of the surgical incision with a drainage tube placed from the assisted hole. (A colour version of this figure appears in the online version of this article.)

The first da Vinci SP RTG was successfully conducted with a 10-min docking time and 245-min intracorporal operation time. The estimated blood loss was 225 mL. Except for the subcutaneous emphysema, no severe adverse events occurred during the operation. We used a visual analogue scale (VAS) to evaluate the subjective feeling of post-operative pain and found a VAS score of 3 of 10 on Post-Operative Day 1 (POD 1), 1 of 10 on POD 3, and 1 of 10 on POD 7. We removed the gastric tube on POD 2 and advised sipping water, a liquid diet, and a soft diet on POD 2, POD 4, and POD 6, respectively. The patient was discharged without any complications on POD 8 and no uncomfortable symptoms were reported on POD 30 on 22 February 2022. The total stomach specimen and the surgical incision are shown in Figure 5. The post-operative pathological results showed that a tumor located in the upper stomach was a poorly differentiated adenocarcinoma that had invaded the serosa with nerve and vascular invasion, while the tumor located in the antrum of the stomach was defined as moderate–poorly differentiated adenocarcinoma that invaded the deep muscular layer. The upper and lower margins were negative without remnant tumor and 41 LNs were retrieved, including 20 metastatic LNs. Four of the No. 10 LNs showed evidence of metastasis, which demonstrated the necessity for dissection. The final pathological stage was pT4aN3bM0 (Stage IIIc).

## Discussion

Since Hashizume *et al.* first reported robotic gastrectomy in 2002 [13], it has gradually become an alternative surgical approach, as shown in many recent randomized–controlled trials [14, 15]. Compared with conventional robotic gastrectomy, reduced-port or even SP robotic gastrectomy, which has emerged in recent years, minimizes surgical trauma but has been difficult for the surgeons to implement [16]. To date, there is no report on the application of the da Vinci SP robotic platform in robotic gastrectomy for gastric cancer. Here, we report the first da Vinci SP RTG and outline the surgical outcomes and technical points for further application.

The assessment of the mesogastric anatomy and the relationship between the adjacent organs, vessels, and LNs can help surgeons to find correct anatomical clearance, ensure surgical safety, and improve the effectiveness of radical lymphadenectomy. Shinohara *et al.* [17] summarized the embryonic development, visceral anatomy, and surgical techniques of the mesogastrium and suggested that mesogastric excision, which consists of removing the mesenteric adipose tissue containing the LNs, could be a standard technique for surgical treatment. It is crucial to expose the fusion fascia, which is located between the mesogastrium and other structures, including the retroperitoneum, greater omentum, and transverse mesocolon [18–20], and the investing fascia, which encloses the embedding parenchymal organs or vasculature in the mesogastrium [21]. In our study, it was convenient to expand the “Holy plane” with the help of tractive effort from Arm 1 and the first assistant, simultaneously achieving adequate lymphadenectomy without unnecessary hemorrhage based on the mesogastric anatomy. In recent years, Kumazu *et al*. developed a deep-learning model to predict the presence of loose connective tissue fibers during robotic gastrectomy. This technique may help surgeons to determine the correct anastomical planes for their procedures [22].

A positive splenic hilar LN (No. 10 LN) is regarded as an independent risk factor for predicting poor survival in gastric cancer [23]. However, whether No. 10 LNs need to be conventionally dissected for proximal advanced gastric cancer remains controversial [24, 25]. Even though the 5th edition of the Japanese Gastric Cancer Guideline [12] states that No. 10 LNs are excluded from D2 LN dissection for non-greater curvature advanced proximal gastric cancer based on the JCOG0110 study [26], it is strongly recommended to dissect No. 10 LNs because of the higher metastatic incidence, with 15.1% for tumors located on the side of the greater curvature [27]. The deep position and complex vascular structure of No. 10 LNs make this region more difficult to dissect, and thus the manipulative stability and local magnified field of the robotic arms could provide a powerful guarantee of surgical safety. Some previous studies have reported that conventional robotic systems could safely conduct spleen-preserving No. 10 LN dissection [28, 29]; however, there is no relevant report for the use of the SP robot in this region. In this case, we performed No. 10 LN dissection using the da Vinci SP platform because multiple tumors had invaded the greater curvature. After detaching the posterior gastric artery, we gradually exposed the trunk of the splenic artery towards the spleen area and then dissected the LNs along the region of the superior and inferior pole regions of the spleen. We achieved a dissection time of 23 min and the estimated blood loss was 8 mL, which may demonstrate the safety and feasibility of the da Vinci SP platform for performing this procedure. During this period, the first assistant is critical for pulling the stomach in the ventral and cephalic directions, allowing the surgeon's three robotic arms to fully focus on the surgical region.

Intraoperative blood loss is one of the most important indexes for evaluating surgical safety. Chen’s study found that RTG resulted in less blood loss than laparoscopic total gastrectomy (LTG) (38.7 vs 66.4 mL, *P *=* *0.042) [30]. This may be due to the lower average number of errors and surgery task load index of RTG, which can achieve stable manipulation. Hikage *et al*. demonstrated that the median blood loss in the RTG group was 32.5 mL, which was comparable to the LTG group among clinical stage I/IIA gastric cancer patients [31]. For patients with advanced gastric cancer, Yang *et al*. also found that the blood loss of the RTG group was significantly lower than that of the LTG group (154.37 ± 89.68 vs 183.77 ± 95.39 mL, *P *=* *0.004) [32]. The estimated blood loss in our case was 225 mL, which is more than that of RTG reported previously but to an acceptable extent. We considered the following reasons for this discrepancy: (i) the electric scissor located in Arm 3 is the main instrument for performing the key steps during LN dissection; compared with the single working surface and good coagulation effect of the ultrasonic knife, electric scissors are more likely to damage the surrounding tissues and cause unnecessary bleeding. When dissecting No. 6 LNs, because of the close location and anatomic variation of the right gastroepiploic vessels and inferior pyloric vessels [33], the assistant could use an ultrasound knife via the assistive hole to safely complete the dissection of this region. (ii) The triangular operating area of the SP robotic arms partially occupied the operational space, leading to a larger working distance between the camera and the robotic arm. Furthermore, the location of the camera needs to be readjusted using the “relocation” pedal to ensure surgical safety because the movement of the three robotic arms might obstruct the surgeons’ view [34].

When choosing the appropriate patients for robotic gastrectomy, BMI is a key factor in determining the surgical difficulty and short-term outcomes. Strong *et al.* found that a BMI of ≥31 kg/m^2^ was an independent risk factor for conversion to open surgery during robotic gastrectomy [35]. Cong’s study suggested that patients with a BMI of ≥24 kg/m^2^ who underwent RTG required a longer operation time and incurred greater post-operative costs [36]. A retrospective study enrolling 817 patients who underwent robotic gastrectomy showed that a BMI of ≥25 kg/m^2^ was an independent risk factor for systemic complications after robotic gastrectomy [37]. We believe these findings are due to the greater amount of abdominal adipose tissue in patients with a high BMI, which makes it more difficult to find important vessels during the operation and achieve adequate LN dissection, especially for LNs situated at the greater curvature of the stomach [38]. Additionally, a greater amount of visceral adipose tissue is more likely to cause post-operative complications, such as anastomotic leakage, pancreatic-related infection, and abdominal abscess [39]. Thus, we strongly consider patients with a BMI of <24 kg/m^2^ to be suitable for undergoing da Vinci SP robotic gastrectomy for surgeons who are starting to conduct SP robotic surgeries because of the limited large-scale visual field exposure of the SP robot compared with the conventional da Vinci robotic platform.

Some other primary observations also need to be summarized and demonstrated at this point. First, the operation time of the first da Vinci SP RTG is excessively long; surgeons need to cross the learning curve to achieve skilful manipulation. Second, subcutaneous emphysema occurred as an adverse event during surgery in this study, mainly because of the weak abdominal wall of the elderly patient and the inappropriate placement of the 25-mm SP cannula. We suggest that precise design of the incision length for the SP cannula and the development of fixable and anti-infiltration trocars could effectively prevent subcutaneous emphysema. Third, the first assistant needs to help the surgeons to complete complicated procedures, including clamping vessels, division of the duodenum, tissue pulling, hemostasis, etc. only via a 12-mm assistive hole. In addition, when operating in such a narrow space, due to the obstruction of the SP robotic arm, instrument collision may occur when inserting an extra instrument via the assistive hole, which may affect the surgical operation. Therefore, it is recommended to select a surgeon with minimally invasive surgery experience as the assistant during the initial conduction of da Vinci SP robotic gastrectomy. Finally, the equipment could be improved to help promote the clinical application and surgical safety of the SP robot. The wrist-rotatable ultrasonic knife has been proven safe and feasible in animal experiments (Novuson Surgical Inc., USA) and is expected to be applied to the da Vinci SP robotic system as an instrument for use in humans.

In conclusion, we reported the first case of RTG for advanced gastric cancer patients via the da Vinci SP robotic platform and successfully performed D2 LN dissection, including that of the No. 10 LNs, illustrating the safety and feasibility of this technique. In the future, more clinical studies are needed to verify the safety of this operation and the potential value of the boarding application.

## Supplementary Data

Supplementary data is available at *Gastroenterology Report* online.

## Authors’ Contributions

B.W. and L.C. designed this study and performed the operation; H.C., K.C.Z., J.H., and W.Q.L. collected the perioperative data and operative images; H.C. wrote this manuscript and S.Y.L. polished this manuscript; J.X.C. edited the surgical video; B.W. and L.C. supervised the manuscript. All authors have read and approved the final version of the manuscript.

## Funding

This work was supported by the National Basic Research Program of China (973 Program) [grant number 2019YFB1311505] and the National Natural Science Foundation of China [grant number 82073192].

## Supplementary Material

goac023_Supplementary_Data
