# Supplementary material for: Can a single-port robot be safely used for robotic total gastrectomy for advanced gastric cancer? First experience using the da Vinci SP platform
Source: Gastroenterol Rep (Oxf). 2022 Jun 7;10:goac023. doi: 10.1093/gastro/goac023 (PMC9172626; doi:10.1093/gastro/goac023)
Supplement: goac023_Supplementary_Data [file goac023_supplementary_data.zip › Supplement Video ttile.docx]

D2 lymphadenectomy during the single-port robotic total gastrectomy using *da Vinci* SP platform
